# A High-Temperature Piezoresistive Pressure Sensor with an Integrated Signal-Conditioning Circuit

**DOI:** 10.3390/s16060913

**Published:** 2016-06-18

**Authors:** Zong Yao, Ting Liang, Pinggang Jia, Yingping Hong, Lei Qi, Cheng Lei, Bin Zhang, Jijun Xiong

**Affiliations:** 1 National Key Laboratory for Electronic Measurement Technology, North University of China, Taiyuan 030051, China; yaozong126@sina.com (Z.Y.); pgjia@cqu.edu.cn (P.J.); hongyingping_2014@163.com (Y.H.); qilei19850224@163.com (L.Q.); leichengnuc@163.com (C.L.); zb0003@126.com (B.Z.); 2 Key Laboratory for Instrumentation Science & Dynamic Measurement, North University of China, Ministry of Education, Taiyuan 030051, China

**Keywords:** SOI, high-temperature piezoresistive pressure sensor, temperature compensation, integrated signal-conditioning circuit

## Abstract

This paper focuses on the design and fabrication of a high-temperature piezoresistive pressure sensor with an integrated signal-conditioning circuit, which consists of an encapsulated pressure-sensitive chip, a temperature compensation circuit and a signal-conditioning circuit. A silicon on insulation (SOI) material and a standard MEMS process are used in the pressure-sensitive chip fabrication, and high-temperature electronic components are adopted in the temperature-compensation and signal-conditioning circuits. The entire pressure sensor achieves a hermetic seal and can be operated long-term in the range of −50 °C to 220 °C. Unlike traditional pressure sensor output voltage ranges (in the dozens to hundreds of millivolts), the output voltage of this sensor is from 0 V to 5 V, which can significantly improve the signal-to-noise ratio and measurement accuracy in practical applications of long-term transmission based on experimental verification. Furthermore, because this flexible sensor’s output voltage is adjustable, general follow-up pressure transmitter devices for voltage converters need not be used, which greatly reduces the cost of the test system. Thus, the proposed high-temperature piezoresistive pressure sensor with an integrated signal-conditioning circuit is expected to be highly applicable to pressure measurements in harsh environments.

## 1. Introduction

In recent years, silicon piezoresistive pressure sensors fabricated using MEMS technology have been extensively applied in commercial and industrial fields because of their small size, high precision and low cost [1]. Additionally, there is a growing demand for high-temperature pressure sensors for use in extreme temperature environments such as oil prospecting, chemical processing and aircraft gas turbine combustion controls, where pressure sensors must usually work at 220 °C or higher [2]. The high-temperature applications of silicon piezoresistive pressure sensors have become a current research priority.

PN-junction isolation technology among piezoresistive strain elements is used in traditional pressure sensors that are based on bulk silicon production. As the temperature increases, PN-junction reverse-leakage current increases, leading to short circuits between elements and device failure. Therefore, the pressure sensors’ working temperature with PN-junction isolation technology does not exceed 125 °C [3]. Recently, with the use of dielectric isolation technology materials, silicon on insulation (SOI), and wideband-gap semiconductor material SiC, GaN, the working temperature of piezoresistive pressure sensors can reach 300 °C or even higher [4]. However, the microfabrication technology of wideband-gap sensor materials is far less mature than silicon micromachining technology. Further progress must be made before these wideband-gap sensors can become cost-effective products. Therefore, the development of high temperature low-cost micromachined silicon piezoresistive transducers based on SOI technology remains attractive.

**Figure 1:**
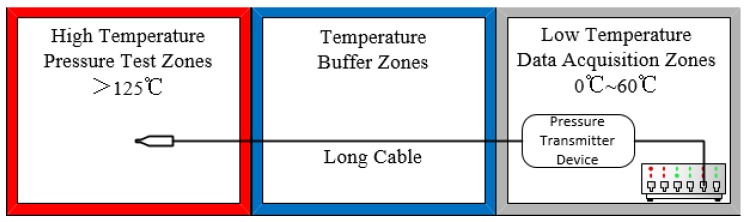
High-temperature pressure sensor application installation diagram.

Compared to general pressure sensors in the temperature range used in industry, high-temperature pressure sensors are usually installed in high-temperature pressure test zones; their output signals are sent to low-temperature data acquisition zones via long cables through temperature buffer zones [5], as shown in Figure 1.

**Figure 2:**
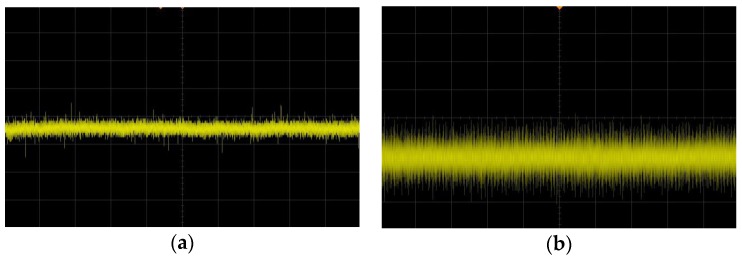
Sensor signal transmission interference comparison chart (**a**) direct output signal; (**b**) output signal after 5-m-long cable transmission.

There are more high-temperature thermal noises, electromagnetic noises and other interference factors than in conventional application environments [6]; therefore, more consideration should be given to improving the signal-to-noise ratio (SNR) among the sensor optimization measures [7]. However, signal interference is inevitable over long distances, especially for the signal output of piezoresistive pressure sensors, which usually has a small output range (from tens to hundreds of millivolts). This interference has a serious impact on measurement accuracy. As shown in Figure 2, after 5-m-long cable transmission for the full-scale output of a 100 mV piezoresistive pressure sensor, measurement accuracy decreases from 1% to 3% because of signal interference.

Unlike the general high-temperature pressure sensor with a single chip based on SOI technology [8], the proposed device, integrated with signal-conditioning circuits, can convert a small signal output to a 0 V to 5 V standard voltage signal output, which can significantly reduce the ambient noise interference over long cable transmissions and improve the measurement accuracy. Additionally, general follow-up pressure transmitter devices in the voltage converter (shown in Figure 1) need not be added, because the standard signal output can be directly acquired by the signal acquisition system, which greatly reduces the cost of the test system.

## 2. Design and Fabrication of the High-Temperature Piezoresistive Pressure Sensor

### 2.1. Design and Fabrication of the Pressure-Sensitive Chip

(1) Design of the pressure-sensitive chip

**Figure 3:**
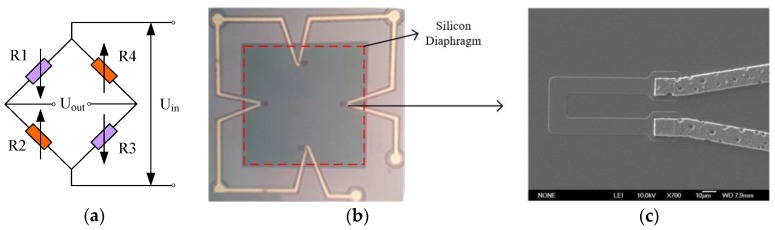
The pressure-sensitive chip’s equivalent circuit and structure (**a**) Wheatstone bridge circuit diagram; (**b**) plan view of the chip; (**c**) enlarged view of the piezoresistor.

The pressure-sensitive chip testing method uses the principle of the Wheatstone bridge, as shown in Figure 3a. The design parameters include the doping concentration; the shape and size of the pressure-sensitive chip and its diaphragm; and the size, shape, and placement of piezoresistors.

Based on the relationship curve of doping concentration, the temperature coefficient of the piezoresistor (TCR), and the temperature coefficient of the piezoresistive coefficient (TCπ) [9], the doping concentration value of 2 × 10^18^ cm^−3^ is used for the production of a boron-doped device layer, in order to make sensitivity temperature drift as small as possible. The design of a 2 mm side-length square structure is used for the pressure-sensitive chip for ease of processing, cost reduction, and increased production (each piece of the SOI wafer can produce no less than 1300 sensitive chips).

For ease of manufacture, the silicon diaphragm is a sensitive membrane in the form of a square, as shown in Figure 3b. By including the existing theoretical foundation, we found that a sensitive silicon diaphragm of thickness h with side length a should meet the following three requirements: first, output linearity requires that the sensitive diaphragm should follow the small deflection theory model, which means that the maximum deflection of the diaphragm is less than 20% of the thickness of the center; second, the maximum overload stress requires that the combined stress value (the difference of longitudinal stress and transverse stress) at each point on the film is less than or equal to 30% of the material rupture stress; third, the higher sensitivity output requires that the relative rate of change of the varistor be greater than 2%. According to the shell theory formula [10], the requirements above can be restated by Equation (1):

(1) $$\left\{ \begin{array}{l} w_{\max}=0.01518\frac{pa^{4}\left(1-\upsilon^{2}\right)}{Eh^{2}}\leq 20\%h\\ \max(\left|\sigma_{l}-\sigma_{t}\right|)=0.308P_{\max}\frac{a^{2}}{h^{2}}(1-\upsilon )\leq 0.3\sigma_{m}\\ \frac{\Delta R}{R}=\frac{0.308}{2}\pi_{44}P\frac{a^{2}}{h^{2}}(1-\upsilon )\geq 2\% \end{array}\right.$$

where the piezoresistive coefficient π_44_ = 138 × 1011 Pa^−1^, Young’s modulus E = 190 GPa, Poisson’s ratio v = 0.28. When the pressure sensor range P = 2 MPa, pressure can be calculated from the equation set for a reasonable range of a/h from 22 to 28. For the side length of 2 mm of the pressure-sensitive chip, the thickness of the diaphragm can be set to 40 μm for a sensitive diaphragm side length of 1 mm.

The four piezoresistors size should meet the components’ power requirement that the rated power per unit area of the components is less than the dissipated power per unit area of the material. Otherwise, the accumulation of heat easily causes the components to undergo fuse failure. The power dissipated per unit area of silicon is 5 × 10^−3^ mW/μm^2^ [11], which can be expressed as:

(2) $$P_{c}=\frac{I^{2}R_{P}}{\text{WL}}=\frac{I^{2}R_{S}\frac{L}{W}}{\text{WL}}=\frac{I^{2}R_{S}}{W^{2}}$$

where R_P_ is the piezoresistor’s resistance value, R_S_ is the square resistance, W is the resistance strip width, L is the resistance strip length, and I is the operating current value. Based on the target square resistance, the constant voltage source power supply, and the derating, the size of the resistance strip can be set to 10 μm × 150 μm, with a “U”-shaped structure, as shown in Figure 3c.

**Figure 4:**
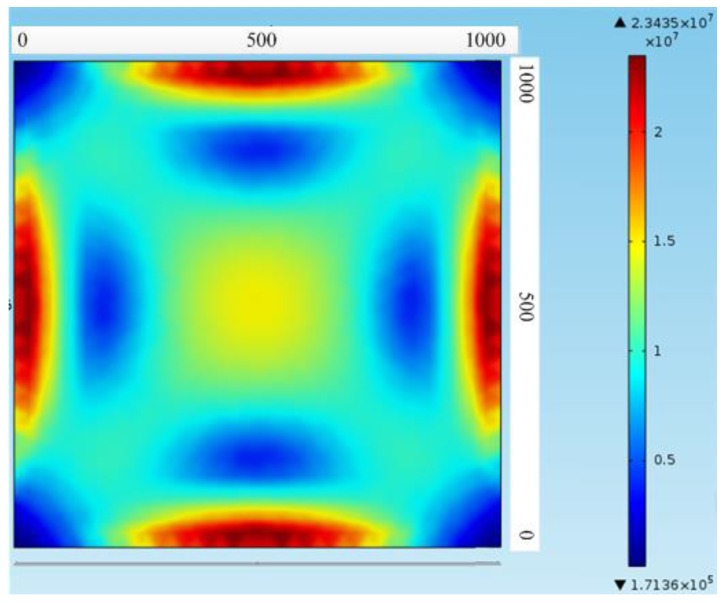
Pressure-sensitive chip simulation in stress distribution diagram.

Figure 4 shows the pressure-sensitive chip simulation of stress distribution diagram. It can be obviously seen that stress concentration areas are an intermediate position of the four edges of the sensitive diaphragm. Therefore, the piezoresistors should be placed in stress concentration areas to improve the sensitivity of the sensor.

(2) Fabrication and package of the pressure-sensitive chip

**Figure 5:**
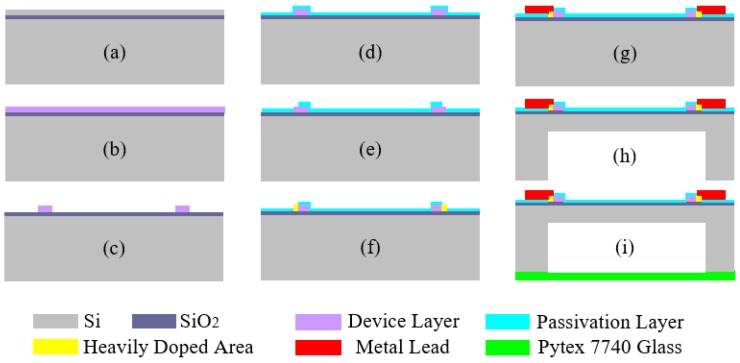
Pressure-sensitive chip production flow chart.

The SOI material and the standard MEMS process is used in pressure-sensitive chip fabrication, as shown in Figure 5.

a) SOI wafer: 4 inches, n-type, <100> crystal orientation, silicon device layer thickness of 2 μm, silicon dioxide dielectric spacer layer thickness of 1 μm, total thickness of 400 μm.
b) Boron doping: Solid source of boron thermal diffusion process is used for boron doping in the silicon device layer, at a target doping concentration of 2 × 10^18^ cm^−3^.
c) Etching piezoresistors: an inductive coupled plasma (ICP) dry etching process is used to etch piezoresistors on the silicon device layer.
d) Depositing a passivation layer: the plasma enhanced chemical vapor deposition (PECVD) process is used to deposit a passivation layer with a total thickness of 0.3 μm of SiO_2_ and Si_3_N_4_ so as to protect the piezoresistors.
e) Etching the ohmic contact region: the ICP dry etching process is used again to etch the ohmic contact region between the piezoresistors and the metal lead.
f) Heavy doping in the ohmic contact region: a solid source of boron thermal diffusion processes is used again to heavily dope the ohmic contact region as to reduce the contact resistance between the piezoresistors and the metal lead, with a target doping concentration greater than 2 × 10^20^ cm^−3^.
g) Sputtering metal lead: the sputtering process is used to produce a metal lead and wire bonding pads. The three sputtered layers of metal are Ti, Pt, and Au from bottom to top; among them, Ti is an adhesion layer, Pt is a barrier layer, and Au is a conductive passivation layer. To ensure the follow-up wire bonds reliably, the thickness of the Au layer should be greater than 200 nm.
h) Release the pressure-sensitive diaphragm: the BOSCH process is used to deeply etch the bottom of the wafer, creating a pressure-sensitive diaphragm.
i) Making the reference absolute pressure chamber: the anodic bonding process between the bottom area of wafer and 300-um-thick glass Pyrex 7740 is used to make an absolute pressure reference chamber.

**Figure 6:**
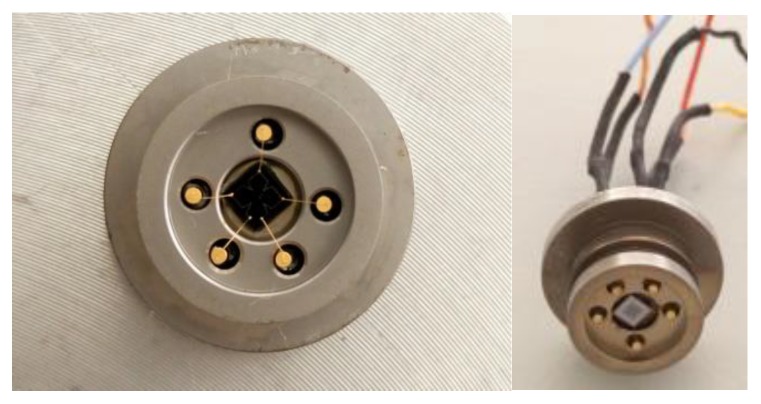
The pressure-sensitive chip modular package.

The pressure-sensitive chip (glass surface down) is mounted on a 316 L stainless steel base using a high-temperature adhesive to ceate a modular package, as shown in Figure 6. The electrode pads of the pressure-sensitive chip are leaded to Kovar pins of the package base using a thermo-compression wire bonding process. Insulating glass is filled into the gap between the Kovar pins and the package base to ensure good electrical isolation.

### 2.2. Design of the Pressure-Sensitive Chip Temperature Compensation Circuit

For high-temperature piezoresistive pressure sensors following the principles of Wheatstone bridge measurement, continuous drift in the sensor’s output voltage is inevitable, because the piezoresistance increases with temperature, whereas the piezoresistive coefficient decreases [12]; this phenomenon adversely affects the testing precision. Therefore, it is critical to achieve effective temperature compensation for high-temperature piezoresistive pressure sensors. Commonly used temperature compensation methods include hardware compensation and software compensation [13]. Software compensation requires a large number of experiments using the pressure-sensitive chip, establishing temperature drift data, then completing compensation using an inverse function algorithm or an artificial neural networks algorithm [14], which usually has extremely high accuracy; however, the complex compensation system and the high cost of testing make it difficult to achieve large-scale production. In contrast, hardware compensation, because of the characteristics of simplicity, reliability, low cost, high accuracy, wide application range, and ease of batch manufacturing, has been widely used. Hardware compensation typically uses an additional thermistor, a low temperature coefficient resistor network, a diode, a triode, an adjustable gain operational amplifier, and so on [15].

**Figure 7:**
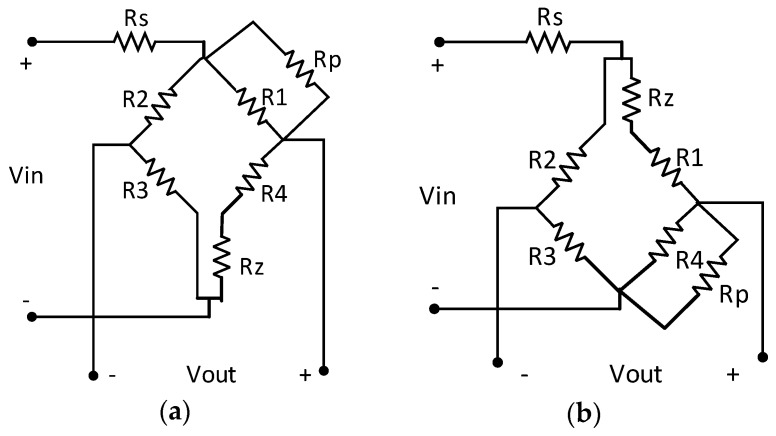
Passive resistor temperature compensation model with a constant voltage supply: (**a**) compensation model for negative initial offset voltage; (**b**) compensation model for positive initial offset voltage.

This design of the pressure-sensitive chip temperature compensation circuit uses a passive resistor temperature compensation model based on hardware compensation of low-temperature-coefficient resistor networks, as shown in Figure 7. The bridge circuit is powered by a constant voltage source Vin, with passive compensation resistors Rs, Rp, and Rz. The premise of the model is that the temperature coefficient of the passive resistor should be less than 1% of that of the bridge arm resistor. In this case, the temperature coefficient of the passive resistor can be ignored. Because the temperature coefficient of a typical silicon piezoresistor is greater than 2000 ppm [16], the temperature coefficient of the passive resistor should be less than 200 ppm.

For the negative initial offset voltage of this pressure-sensitive chip, the compensation model in Figure 7a can be adopted, the output voltage expression of which is:

(3) $$\begin{array}{l} V_{\text{OUT}}(T,P)=V_{\text{IN}}\times \frac{{[R}_{2}(T,P)+R_{3}(T,P)]∥{[R}_{Z}+R_{4}(T,P)+R_{1}(T,P)∥R_{P}]}{{[R}_{2}(T,P)+R_{3}(T,P)]∥{[R}_{Z}+R_{4}(T,P)+R_{1}(T,P)∥R_{P}]+R_{S}}\times \\ \left[\frac{R_{Z}+R_{4}(T,P)}{R_{Z}+R_{4}(T,P)+R_{1}(T,P)∥R_{P}}-\frac{R_{3}(T,\text{ P})}{R_{2}(T,\text{ P})+R_{3}(T,\text{ P})}\right] \end{array}$$

The measurement parameters of the compensation model, which should be tested in advance, involve the four bridge arm resistances at the two compensation temperature thresholds of the high-temperature pressure sensor, T_0_, and T_1_ (T_0_ < T_1_), and two load pressures, P_0_, and P_1_ (P_0_ < P_1_). The test results for the bridge arm resistors at temperature thresholds of 20 °C (T_0_) and 220 °C (T_1_) and the load pressures at 200 kPa (P_0_) and 600 kPa (P_1_) are listed in Table 1.

According to the demands of bridge temperature compensation, the algorithm for passive resistor temperature compensation can be written as:

(4) $$\left\{ \begin{array}{l} V_{\text{OUT}}(T_{0},\;P_{0})=U_{0}\text{ Compensation of offset voltage }U_{0}\\ \frac{\partial V_{\text{OUT}}(T,\;P_{0})}{\partial T}=0\text{ Compensation of temperature coefficient of offset}\\ \frac{\partial V_{\text{OUT}}(T,\;P_{1})}{\partial T}=0\text{ Compensation of temperature coefficient of sensitivity} \end{array}\right.$$

These equations show that the compensation of the temperature coefficient of the offset requires the sensor output voltage under the initial load pressure P_0_ to be independent of temperature. The compensation of the temperature coefficient of sensitivity also requires the sensor output voltage under the higher load pressure P_1_ to be independent of temperature. These requirements mean that the partial derivatives of V_OUT_(T, P_0_) and V_OUT_(T, P_1_) with respect to temperature T must remain equal to zero.

**Figure 8:**
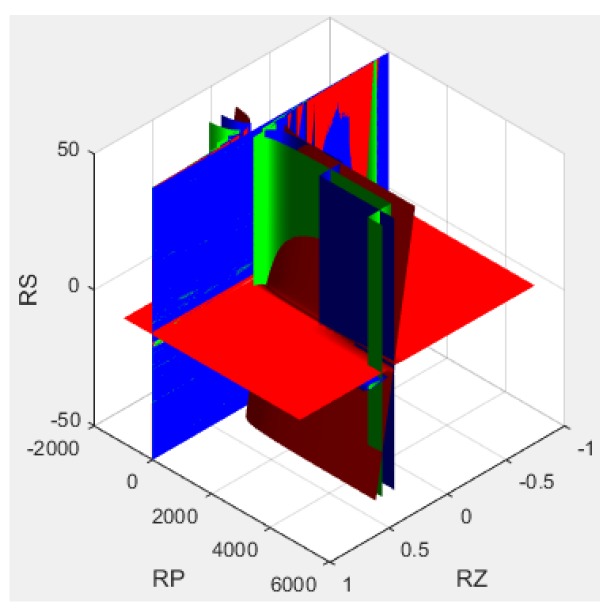
Solving equations by plotting the parameter space in MATLAB.

The algorithm for the passive resistor temperature compensation shown in Equation (4) can be solved by examining the plot in Figure 8, which applies MATLAB to the test data in Table 1 (setting the offset output voltage U_0_ = 4 mV).

The parameter values were varied within the following ranges of compensation resistance:

$$R_{Z}\in \left[0,200\Omega \right],\;R_{P}\in \left[1k\Omega ,1000k\Omega \right],\;R_{S}\in \left[1k\Omega ,30k\Omega \right]$$

From these parameters, we obtained the minimal compensation resistance parameters:

$$R_{Z}=110\Omega ,\;R_{P}=180k\Omega ,\;R_{S}=20k\Omega$$

**Figure 9:**
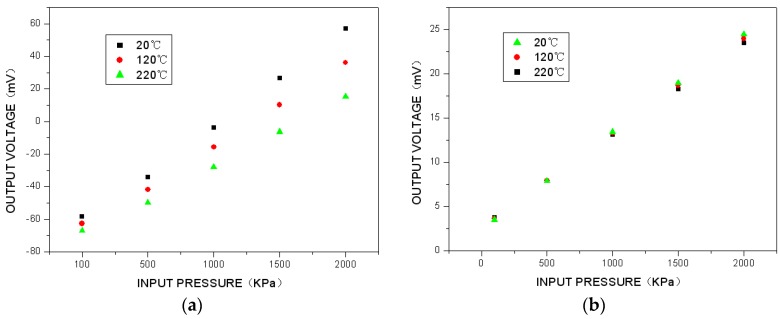
Comparison of pressure-sensitive chip temperature compensation: (**a**) uncompensated sensor calibration curve in temperature and pressure environment; (**b**) compensated sensor calibration curve given by the passive resistor temperature compensation in a high-temperature and high-pressure environment.

Sensor calibration was conducted for uncompensated and compensated sensors in the range of 20–220 °C and 100–2000 kPa. The results are shown in Figure 9. The uncompensated sensor calibration curve (Figure 9a) shows significant variation over the temperature range; the overall accuracy is ±18%FS. However, the sensor calibration curve compensated using the passive resistor temperature compensation (Figure 9b) exhibits significantly better accuracy at ±1.5%FS over the entire temperature range.

### 2.3. Design of High-Temperature Signal-Conditioning Circuit

The main function of the high-temperature signal-conditioning circuit is amplification of the sensor output voltage signal from tens of millivolts to 0 V~5 V. Additionally, the circuit has a flexible adjustable range in offset voltage and magnification. A high temperature operational amplifier (HT1104) and a linear regulator (HTPLREG05) produced by Honeywell (Morris Plains, NJ, USA) are used; these devices have long-term stability working in the temperature range from −55 °C to +225 °C.

**Figure 10:**
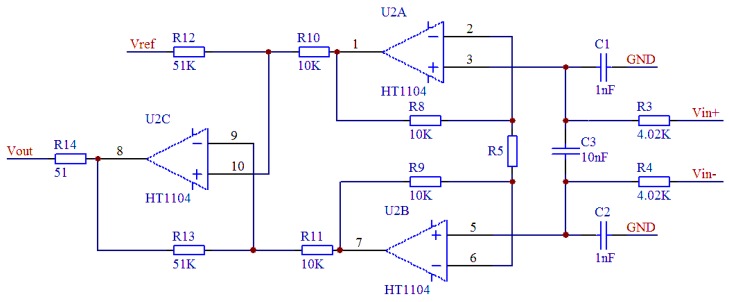
High-temperature signal-conditioning circuit schematic.

Based on the high output impedance of the pressure-sensitive chip, a typical value in the range 1 kΩ to 8 kΩ due to materials and process errors, the high-temperature signal-conditioning circuit should have a high input impedance. Here, three operational amplifiers are used to improve the circuit input impedance, as shown in Figure 10.

The pressure-sensitive chip’s positive and negative outputs can be connected to the two operational amplifiers’ non-inverting inputs (Vin+ and Vin−) to ensure a high input impedance. To prevent signal amplifying circuit zero voltage drift due to RF rectification effects [17], an RC low-pass filter network consisting of C1 to C3, and R3 to R4 (C1 = C2, C3 = 10C1) can be used to eliminate RF interference. The differential mode and common mode filter cut-off frequencies, which are adjustable based on the pressure-sensitive chip frequency range, can be expressed as:

(5) $$\begin{array}{l} f_{\text{DIFF}}=\frac{1}{2\pi R(2C_{1}+C_{3})}\\ f_{\text{CM}}=\frac{1}{2\pi {\text{RC}}_{3}} \end{array}$$

Vref for DC bias voltage of the output amplified signal can be obtained by linear regulator output voltage division. R5 (the gain adjustment resistor) determines the output voltage of the high-temperature signal-conditioning circuit, which can be expressed as:

(6) $$V_{\text{OUT}}=5.1\times (1+\frac{20K}{R5})\times (V_{\text{in}+}-V_{\text{in}-})+V_{\text{ref}}$$

### 2.4. Design of the Sensor Structure and Its Assembly

**Figure 11:**
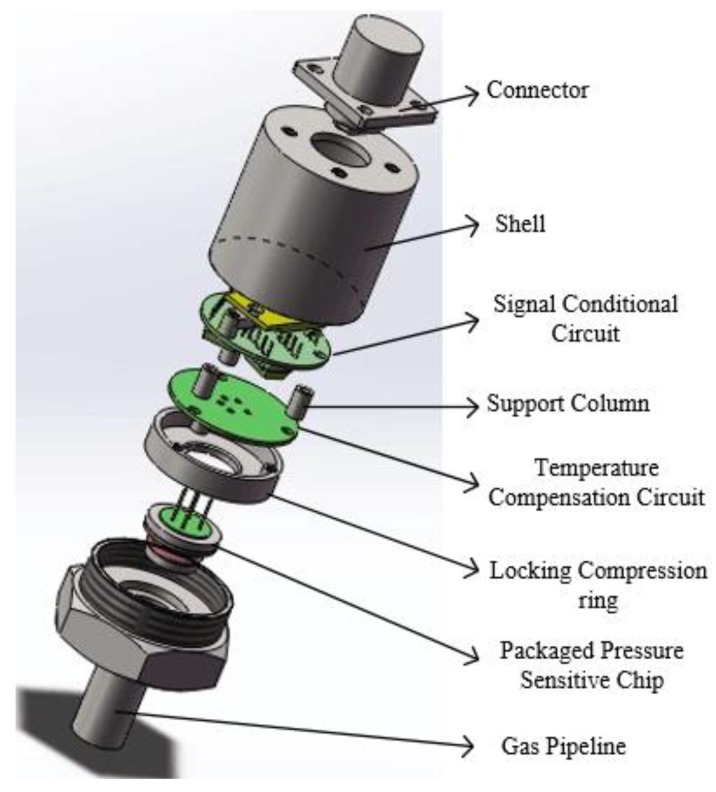
Assembly structure of high-temperature piezoresistive pressure sensor with integrated signal-conditioning circuit.

**Figure 12:**
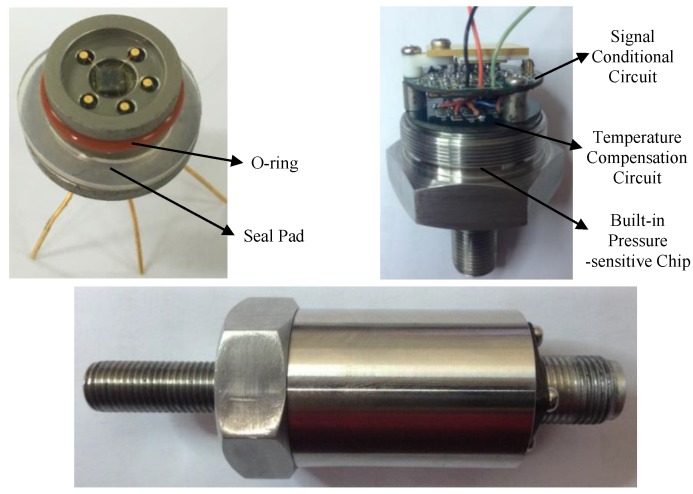
Sensor device pictures.

The assembly structure and pictures of the high-temperature piezoresistive pressure sensor with integrated signal-conditioning circuit are shown in Figure 11 and Figure 12, respectively. The device has the characteristics of small size, light weight, easy dismantling, and good sealing performance. The pressure sensor adopts a bottom-up vertical assembly process. At the beginning, the packaged pressure sensitive chip is mounted in gas pipeline with seal rings and is fastened to the end by the locking compression ring, which achieves a hermetic seal. Then, the temperature compensation circuit and the signal-conditioning circuit are successively installed using the support column and the screws fastening; the electrical interconnections among them are created using a high-temperature cable. Finally, the shell and connector are assembled to export the signal and the power supply interface.

## 3. Sensor Performance Test

**Figure 13:**
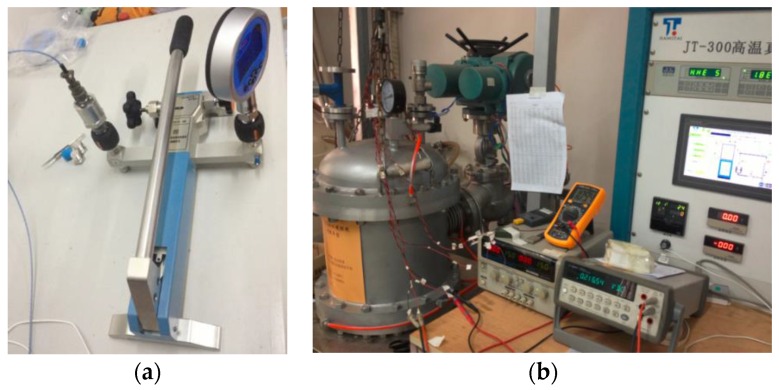
Calibration devices for pressure sensors: (**a**) pressure calibration device at room temperature; (**b**) high-temperature and pressure calibration device.

**Figure 14:**
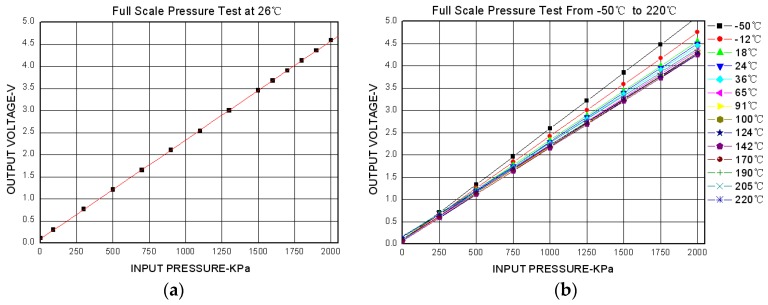
Pressure sensor calibration test results: (**a**) full scale pressure test at 26 °C; (**b**) full scale pressure test from −50 °C to 220 °C.

The measurement parameters of the high-temperature piezoresistive pressure sensor with integrated signal-conditioning circuit, have a range of 2 MPa and can be tested using the calibration devices shown in Figure 13; the test results are shown in Figure 14.

The sensor parameters and corresponding performance comparison with XTE-190 (produced by KULITE, Leonia, NJ, USA) are listed in Table 2.

As can be seen, this sensor’s sensitivity is much larger than that of the XTE-190, which is beneficial for test applications, and the total accuracy in the compensation temperature range is slightly lower than that of the XTE-190 (however, this can be further improved by reducing parameters drift at high temperatures, which is due to the pressure sensor residual stress).

**Figure 15:**
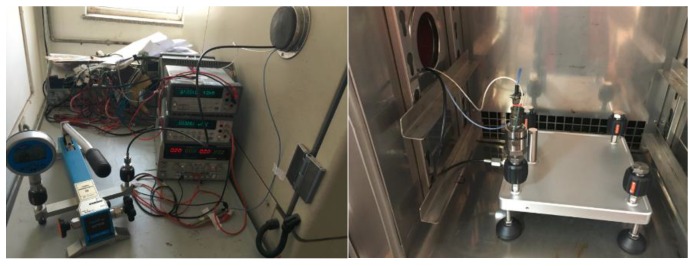
Pressure sensor reliability test.

Reliability testing can be carried out through a temperature cycle test and an aging test, as shown in Figure 15. After the aging test (operating for 40 h at 150 °C), and the ten temperature cycle test where the sensor operates for 1 h at (alternately) −50 °C and 220 °C, the rate of temperature change is 10 °C/min, and the parameters of the sensor do not change. The estimated time zero drift and time sensitivity drift are less than 1%FS.

The results of the 5 m long-term transmission comparative test between the high-temperature piezoresistive pressure sensor with an integrated signal-conditioning circuit and the device without it show that the SNR of the sensor with an integrated signal-conditioning circuit can be improved by more than 20 dB, which suggests significant advantages for long-term transmission applications.

## 4. Conclusions

This paper focuses on the design and fabrication of a high-temperature piezoresistive pressure sensor with an integrated signal-conditioning circuit, which consists of an encapsulated pressure-sensitive chip, a temperature compensation circuit, and a signal-conditioning circuit. The SOI material and the standard MEMS process are used in pressure-sensitive chip fabrication, which adopts the base package for ease of installation; high-temperature electronic components are adopted in the temperature compensation and signal-conditioning circuits. The entire pressure sensor achieves a hermetic seal. The output temperature drift of the pressure sensor is corrected by a passive resistor temperature compensation model whose parameters can be solved using an algorithm based on the calibration data of the uncompensated pressure-sensitive chip. Using the temperature compensation circuit and the signal-conditioning circuit, the small output signal of the pressure-sensitive chip is amplified into 0 V to 5 V (which is a standard voltage output signal), which can be certified to have better signal-to-noise ratio in a long-term transmission test. Additionally, because this flexible sensor output voltage is adjustable, the general follow-up pressure transmitter devices for the voltage converter need not be used, which greatly reduces the cost of the test system. Thus, the proposed high-temperature piezoresistive pressure sensor with an integrated signal-conditioning circuit is expected to be highly applicable to pressure measurements in harsh environments.

## Figures and Tables

**Table 1:** Test results for bridge arm resistors under different environmental conditions.

Conditions	R1 (kΩ)	R2 (kΩ)	R3 (kΩ)	R4 (kΩ)
(20 °C, 200 kPa)	4.53	4.433	4.795	4.695
(20 °C, 600 kPa)	4.529	4.474	4.793	4.743
(220 °C, 200 kPa)	6.777	6.628	7.1698	6.975
(220 °C, 600 kPa)	6.75	6.654	7.159	7.016

**Table 2:** Performance comparison of similar sensor parameters.

Parameters	The Proposed Sensor	XTE-190
Operational mode	Absolute	Absolute
Pressure range	2 MPa	1.7 MPa
Compensated temperature range	+20 °C ~ +220 °C	+25 °C~+232 °C
Sensitivity (10-V power supply)	210 mV/100 kPa	8 mV/100 kPa
Combined non-linearity, hysteresis and repeatability	± 0.5%FSO	± 0.5%FSO
Total accuracy in compensation temperature range	±2%FS	±1.5%FS
